# Brexit in the channel: Europe cut off: a young German scientist’s perspective

**DOI:** 10.1007/s00430-019-00646-1

**Published:** 2019-11-29

**Authors:** Andreas R. Kiessling

**Affiliations:** Astbury Centre for Structural Molecular Biology, School of Biomedical Science, University of Leeds, Leeds, LS2 9JT England, UK

I am writing this just before the end of March and still each option for Brexit seems just as likely as any other. Even if the UK remains in the EU, the chasm in British society, which became apparent in the 2016 referendum, will further grow [1]. That is what the papers say and that is what I experience as I walk the streets of Leeds with the homeless—local, white English men and women—on every corner. It is very different to the small town I grew up in, where we had one homeless person whom everyone knew and liked.

I was born in the 90s into a unified Germany and an ever-growing European Union. Our generation experienced the biggest jump in technology so far with social media connecting people all over the world. From the start, I felt more European than German and at every stage of my life, visa-free travel made short trips and holidays inside the EU a carefree experience. In 2004, I was just 12 years old, when the largest expansion occurred, enabling easy travel through part of the former Eastern Block; it really seemed that we were living at the “end of history” as Fukuyama put it in 1992 [2]. How times have changed!

As a newly minted Biochemistry student, the Erasmus+ programme in 2018 gave me the freedom to study in the UK to learn new techniques and experience a new culture. From the moment I set foot on Shakespeare’s “sceptre’d isle/This earth of majesty”, it was clear that things here ran differently than in the other countries I had visited in Europe. Theresa May had just said “If you believe you are a citizen of the world, you are a citizen of nowhere”—effectively disenfranchising my bosses and people like me. Unlike in the rest of Europe, I noticed a distinct lack of the enthusiasm you find amongst the young for the European project. This feeling is in contrast to recent polls suggesting similar levels of identification as a European citizen among young British people as seen in Germany [3].

My friends and family in Germany kept asking me how could this Brexit mess have happened. In the beginning I could not give them a good answer but, over the last year, I have gained a lot of insight into the bitter divides that exist in British society. They are partly due to the decline of influence of the UK on the former Commonwealth nations, but mostly due to the effects of globalism and the widening gap between rich and poor [4]. The scapegoat for the negative effects of globalism has been the European Union for the UK, but the refugee crisis for Germany. Both expose an underlying xenophobia and a desire for the past, but without having to abandon smart phones and cheap food, social media and fast fashion. Both events expose the separations between rich and poor, old and young, mobility and community that have been growing for more than a decade now. Their resolution will likely take another generation.

I was one of the few people in my year that still considered going to the UK after the 2016 vote and even though I planned to return before Brexit, the warnings and fears of my friends and family were plentiful. In my naivety, I thought that Brexit would be eventually averted and everything would be fine. Even when I accepted a PhD position in the UK in 2018, I was not worried that anything would change for me even though I would be in the UK after the set leaving date in March 2019. A lot has changed since then. It started with small things like having to apply for so-called “settled status”, but suddenly I found myself being afraid of not being able to finish my PhD in the UK, while our lab is stockpiling consumables from Europe and my British friends are buying 2 months’ worth of canned baked beans in the event of a “No-deal” Brexit. Why a country would voluntarily want to put itself on a war-footing, where it is not certain that there will be enough *Insulin* boggles the mind.

Apart from the personal side of events, the effect on UK science looks set to be devastating as collaborations with the rest of the EU are essential for both UK and European science. The Wellcome Trust, one of Britain’s most influential funding bodies experienced a rapid decline in applicants for their PhD programmes from outside of the UK. This also extends to the Erasmus and Marie Curie schemes, with funding unclear after 2020 [5]. The facts are clearly on the side of free travel when it comes to high-impact research [6]. The Nobelist Paul Nurse said that on left and right “Leaders have sleepwalked the nation into what I think is a big disaster for science” [7].

In a fact sheet published by the Royal Society in 2018 regarding the impact of a “No-deal” Brexit, the implications of leaving the EU on scientific collaboration especially those from within the Horizon 2020 framework become apparent: For the time being, the UK is one of the leading countries in terms of publication output with 15% of the most highly cited scientific papers coming from the UK, while at the same time having less than 1% of the world’s population. 33.5% of UK research papers have been co-authored by other EU member states and countries inside the Horizon 2020 funding scheme, which profoundly shows the importance of the UK–EU collaboration compared to the US as second biggest collaborator [8]. This is only the tip of the iceberg in what would be possible if we Europeans continue working as close as now. Especially, the training networks that are part of the Horizon 2020 programme provide a framework for what might well be the standard way of how science will function in the future. With scientific instrumentation becoming more expensive and centralised, like synchrotron light sources and cryo-electron microscopy facilities, pan-European networks like Instruct-ERIC for the field of structural biology enable high-impact science without the need for pre-existing collaborations that require time to grow and foster. Suddenly loosing access to these mentioned programmes will severely affect the way UK science will be perceived in the rest of the world. We must not forget that all of this is happening at the same as China is emerging as an economic and scientific superpower. Clearly, we need an even stronger European Union, not a fractured one, to compete with this development.

The need to organise themselves and become politically active has been largely non-existent with young people in Germany and the UK up until recently. Even today, as a percentage, fewer of us vote than our grandparents [9]. There might still be hope for the next generation of students: the #FridaysforFuture movement is one example of revived political engagement in young people. The naivety with which young people engage in topics that were long thought to be lost in conflict might give new answers that my generation and the older generations are unable to see at the moment. I hope that the next generation of scientists grows up in a world in which visa-free travel is fact not fiction, so we might see the day when collaboration is the focus of events, not backwards-driven politics.

Generally speaking, nationalistic approaches can never be sufficient to solve global problems as big as the climate catastrophe or the antibiotic resistance crisis. The latter is of particular interest to me, as this is a development I can actively help to resolve as a biochemist. All the research in that area is meaningless though if governments do not implement guidelines that affect every country equally. After all, we live in an age in which globalism has brought a lot of different countries and societies together. This results in one big pragmatic realisation: Borders are a social construct that do not make halt for infections or climate and environmental changes. In an extreme example of this forced intertwinement of countries, Germany has decided, back in 2011, to shut down all nuclear plants by 2022, while on the other side of the Rhine, nuclear power is still one of the major sources of French energy. In the worst case scenario of a nuclear meltdown, Germany and surrounding countries would be equally affected by the fallout than France would be although being separated by borders and current views on nuclear energy. The same inevitable conjunction applies to the spread of infectious diseases with more people than ever flying to far-away countries [10]. This enables pathogens to spread that were originally an uncommon occurrence in that country. A mere morale imperative to combat infections in other parts of the world is not sufficient enough, which we all unfortunately had to experience during the Ebola crisis between 2014 and 2016. Efforts have been made to increase the preparedness of countries for these outbreaks, but there is still a lack of research funding and in governmental control that needs to be addressed, if we want to continue enjoying the benefits of a globalised world [11]. As part of the EU-funded training network ViBrANT (**Vi**ral and **B**acte**r**ial **A**dhesin **N**etwork **T**raining), my colleagues and I actively contribute to the development of new research strategies to combat infectious diseases. We 15 graduate students come together from countries from all over the world, with different social and scientific backgrounds, in mutual collaboration, sharing a common goal—this is the European spirit that keeps the heart of the EU pumping and this is the inspiration that I hope other people in the EU will eventually experience themselves.

In the meantime, I have to choose each day between manning the barricades and manning the lab bench. As the Chinese curse has it, we live in “interesting times”.

## References

[1] Ford Robert, Goodwin Matthew (2017). A Nation Divided. Journal of Democracy.

[2] Fukuyama F (1992) The end of history and the last man. Free Press. ISBN 978-0-02-910975-5

[3] Rapp C (2019) Youth Study “Young Europe 2019” of TUI Foundation. TUI Care Foundation. https://www.tuigroup.com/en-en/media/press-releases/2019/2019-05-03-tui-foundation-youth-study. Accessed 14 Nov 2019

[4] Hobolt SB (2016). The Brexit vote: a divided nation, a divided continent. J Eur Public Policy.

[5] Butler D (2019). Number of EU research students in Britain drops from pre-referendum high. Nature.

[6] Sugimoto CR, Robinson-Garcia N, Murray DS (2017). Scientists have most impact when they’re free to move. Nature.

[7] Gibney E (2019). Paul Nurse on Brexit: ‘UK is sleepwalking into a disaster’. Nature.

[8] “No-deal” is a bad deal for science. (2018) Royal Society. https://royalsociety.org/-/media/policy/Publications/2018/royal-society-brexit-no-deal-factsheet.pdf. Accessed 14 Nov 2019

[9] Curtis C (2019) UK general election survey 2017. YouGov. https://yougov.co.uk/topics/politics/articles-reports/2017/06/13/how-britain-voted-2017-general-election. Accessed 1 Aug 2019

[10] Findlater A, Bogoch II (2018). Human mobility and the global spread of infectious diseases: a focus on air travel. Trends Parasitol.

[11] Leigh J, Moon S, Garcia E (2018). Is global capacity to manage outbreaks improving? An analysis.

